# Efficient extraction of oil from droplet microfluidic emulsions

**DOI:** 10.1063/1.4984035

**Published:** 2017-05-19

**Authors:** J. R. Haliburton, S. C. Kim, I. C. Clark, R. A. Sperling, D. A. Weitz, A. R. Abate

**Affiliations:** 1 Department of Bioengineering and Therapeutic Sciences, California Institute for Quantitative Biosciences (QB3), University of California, San Francisco, California 94158, USA; 2 Integrative Program in Quantitative Biology (iPQB) Biophysics Graduate Program, University of California, San Francisco, California 94158, USA; 3 Department of Physics and School of Engineering and Applied Sciences, Harvard University, Cambridge, Massachusetts 02138, USA

## Abstract

Droplet microfluidic techniques can perform large numbers of single molecule and cell reactions but often require controlled, periodic flow to merge, split, and sort droplets. Here, we describe a simple method to convert aperiodic flows into periodic ones. Using an oil extraction module, we efficiently remove oil from emulsions to readjust the droplet volume fraction, velocity, and packing, producing periodic flows. The extractor acts as a universal adaptor to connect microfluidic modules that do not operate under identical flow conditions, such as droplet generators, incubators, and merger devices.

## I. INTRODUCTION

Microfluidics is a rapidly advancing field that is transforming multiple scientific disciplines by allowing the precision control of fluids at picoliter scales.^1–3^ Droplet microfluidics is a branch of this field in which a heterogeneous sample is partitioned into millions of distinct aqueous droplets in an immiscible carrier oil.^4–6^ The ability to partition heterogeneous systems into subsamples is amazingly useful for applications across chemistry and biology. For example, when applied to molecules, it enables precise quantitation with digital enzyme-linked immunosorbent assay (ELISA)^7,8^ and polymerase chain reaction (PCR).^9,10^ When applied to cells, it enables extremely high-throughput single-cell analysis, the evolution of enzymes with unnatural properties, and the construction of pathways for biosynthesis of artificial molecules.^11–13^ It can be used to characterize heterogeneous populations of cells and identify rare members, which is valuable in cancer, immunology, and infectious diseases.^14–17^

Most biological reactions require multiple steps of sample purification, incubation, and reagent addition, which are typically accomplished using microfluidic devices for droplet splitting, merging, and sorting.^18–21^ Like any engineered system, microfluidic components have distinct regimes of optimal operation. Key factors that determine the efficiency of these operations are the flow rates, oil volume fraction, and periodicity of droplets. For example, droplet formation typically requires a high fraction of oil, but incubation is most uniform when droplets are packed.^22,23^ Similarly, merger devices and picoinjection work best when droplets are periodic and can be synchronized, which requires close-packed emulsions.^24^ Indeed, the packing of droplets and adjustment of the oil fraction are common needs when connecting microfluidic components together.

The simplest way to pack droplets is to collect the emulsion into an off-chip reservoir and allow them to “cream” due to their buoyancy. The packed droplets can then be reinjected into a second device to perform additional operations such as merging or sorting. While simple, off-chip collection has drawbacks. It is only applicable when the incubation between operations is long enough for emulsion transfer and requires a skilled user. Even then, it is error-prone, with droplets often coalescing due to dust, static charge, and flow through syringes, needles, and tubing. Indeed, even for skilled users, reinjection is unreproducible and the emulsions usually contain merged droplets, which can interfere with device operation and reduce the data quality. A superior alternative would be to extract the oil on-chip to avoid off-chip handling. A method to extract the majority of oil from an emulsion would make it easier to perform disparate microfluidic operations on a single chip.

In this paper, we describe a method to efficiently remove oil from an emulsion using an on-chip microfluidic extractor. This allows close-packing of initial dilute emulsion, making droplet flows periodic. The key design element of the extractor is the linear array of short, narrow microchannels connecting the main droplet flow channel and the oil extraction channel, which enables the removal of the majority of the oil while the droplets remain in the main channel. Similar microstructures have been reported for trapping cells or microparticles,^25,26^ reducing flow rates for long-term droplet incubation,^23^ forming lipid bilayer interface between droplets,^27^ and inducing droplet coalescence for phase separation of emulsions.^28^ However, to the best of our knowledge, the thin microchannel array has never been used to control the regularity of a continuous stream of microdroplets for deterministic manipulation of droplets. The oil extractors previously reported in the literature tend to operate at low flow rates and large droplet sizes where the capillary number is sufficiently small that a simple constant height channel will work. The thin microchannel array in our device is fabricated to be smaller than the droplets both in the height and width such that efficient oil extraction can be achieved with the most useful droplet sizes (<50 *μ*m) and generation rates (>1 kHz) for a high throughput. We use the extractor to synchronize initially aperiodic droplet streams with periodic ones to achieve a pairwise merger. Our oil extractor is a universal adaptor for connecting microfluidic components that do not operate under identical flow and volume fraction conditions.

## II. MATERIALS AND METHODS

### A. Device fabrication

A microfluidic device is fabricated by soft lithography^29^ on a 3-inch silicon wafer (University Wafers). Photomasks are designed using the AutoCAD software (Autodesk, AutoCAD for Mac 2014). The design files are provided in the supplementary material. The key element of the device is a set of 119 oil extraction channels (5 *μ*m tall, 15 *μ*m wide, 100 *μ*m long) with a center-to-center spacing of 40 *μ*m. To facilitate the accurate alignment of 5-*μ*m-tall connecting channels to the rest of the layer's structures, the first mask only contains alignment marks. The multilayer master mold is fabricated using four photomasks as follows: (a) 25-*μ*m-tall alignment marks are spin coated using a SU-8 3025 photoresist (MicroChem), exposed, and developed; (b) 5-*μ*m-tall connecting channels (SU-8 3005) are spin coated, aligned, and exposed; (c) 40-*μ*m-tall drop making channels (SU-8 3025) are spin coated, aligned, and exposed; (d) 90-*μ*m-tall oil extracting channels and the remainder of the device including a large drop maker (SU-8 3025) are spin coated, aligned, and exposed. The layers (b)–(d) are then developed together. Poly(dimethylsiloxane) (PDMS) (Momentive, RTV 615) is mixed at a ratio of 10:1, degassed, and poured onto the master in a petri dish. The PDMS is cured at 65 °C for 2 h and cut out using a scalpel. Inlet and outlet holes are punched with a 0.75-mm biopsy core (Harris, Uni-Core 0.75) to fit tightly polyethylene tubing (Scientific Commodities Inc., PE/2, ID: 0.38 mm and OD: 1.09 mm). The punched PDMS channel slab is bonded to a glass slide by activating with an oxygen plasma for 60 s at 1 mbar in a plasma cleaner (Harrick Plasma, PDC-001) and baked at 65 °C for 1 h for complete bonding. The inner surface of the microchannels is treated with Aquapel to render it hydrophobic for stable droplet generation and flow.

### B. Device operation

For the aqueous phase, phosphate-buffered saline (PBS; pH 7.4) solution is loaded into plastic syringes (BD Luer-Lok syringe with 27G½ needle) and connected to the inlets with PE/2 tubing. For the oil phase, hydrofluoroether (HFE; 3M Novec 7500) containing a 2% (w/w) nonionic fluorosurfactant (RAN Biotechnologies, 008-Fluoro-Surfactant) is loaded into the same type of syringes. Syringe pumps (New Era Pump Systems, NE-501) are used to inject fluids at controlled flow rates. For the experiments shown in Fig. 2, flow rates are set by the syringe pumps to be 100 *μ*l/h for the aqueous phase and 400 *μ*l/h for the oil phase. The oil extraction is controlled by setting the outlet tube (open to the atmosphere) at a fixed height with respect to the microdevice. This way of gravity-based control allows for fast scanning of a wide range of flow rates while monitoring droplet behavior via microscopic observation. However, for better reproducibility and prolonged stable device operation, extraction by a syringe pump is preferred. For the experiment shown in Fig. 4, flow rates are set by the syringe pumps to be 80 and 250 *μ*l/h for aqueous and oil phases, respectively, for making the small droplets; and 400 and 800 *μ*l/h for aqueous and oil phases, respectively, for making the large droplets; oil is extracted using a syringe pump at −220 *μ*l/h operating in the withdrawing mode. Droplet formation is imaged on an inverted microscope using a fast-shutter camera (Unibrain, Fire-i 530b). Images are analyzed using LabVIEW and ImageJ using custom scripts to extract droplet positions and pairing ratios. For the merger experiments, high-speed imaging is used (Vision Research, Miro M110) to quantify the number of droplets merging.

## III. RESULTS AND DISCUSSION

**FIG. 1.**
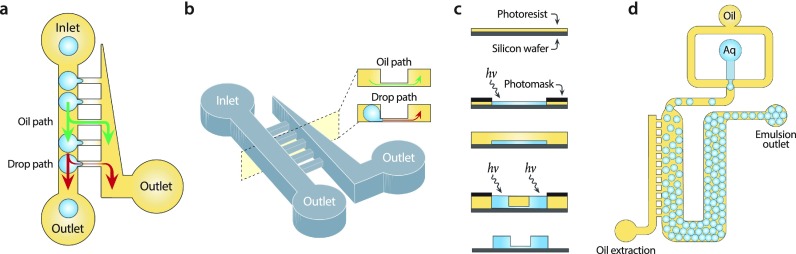
Overview of the oil extractor concept and design. The oil extractor consists of main and extraction channels connected by thin drainage channels. (a) Negative pressure is applied to the extractor outlet, drawing off oil but maintaining the droplets in the main channel due to their inability to deform through the connecting channels. (b) To extract a large fraction of oil while retaining droplets, the connecting channels are narrow and short. (c) The device thus requires two channel heights, which is produced by multi-layer fabrication. (d) By extracting the majority of oil, a dilute emulsion can be packed.

The concept of on-chip oil extraction is to remove the majority of oil from an emulsion while maintaining the droplets inside the channel. A straightforward way to do this is to draw off a controlled portion of oil from the emulsion using narrow channels perpendicular to the main channel. This is possible because for a large droplet to flow through a narrow channel, it must deform. However, deformation increases the Laplace pressure of the droplet, generating a force that opposes entrance into the narrow channel [Fig. 1(a)].^30^ This can be understood via the Laplace law,

$$\Delta P=\gamma \left(1/h+1/w\right),$$

where ΔP is the pressure difference across the droplet interface, γ the interfacial tension, h the height, and w the width. Changing the width and height of a droplet by flowing it into a narrow channel thus increases the pressure in the droplet, allowing it to better resist entrance into the channel. The first oil extractors used channels with the height equal to the main channel but with the narrower width.^23^ While these devices removed some oil, they could not remove the majority of it because to do so requires extracting oil at higher flow rates, but this also extracts droplets. A simple solution would be to increase the Laplace stabilizing force using narrower extraction channels; however, this is difficult with described techniques due to the challenge in fabricating high aspect-ratio channels. Our solution is to reduce the heights and widths of the drainage channels [Fig. 1(b)], which allows a significant increase in the Laplace stabilizing force: While the minimum width of a channel is limited by the resolution of lithographic fabrication, the height is controlled by spin coating [Fig. 1(c)] and can be made reliably below 5 *μ*m; this provides >10× the Laplace stabilizing force and allows extraction of most of the oil from an emulsion [Fig. 1(d)].

**FIG. 2.**
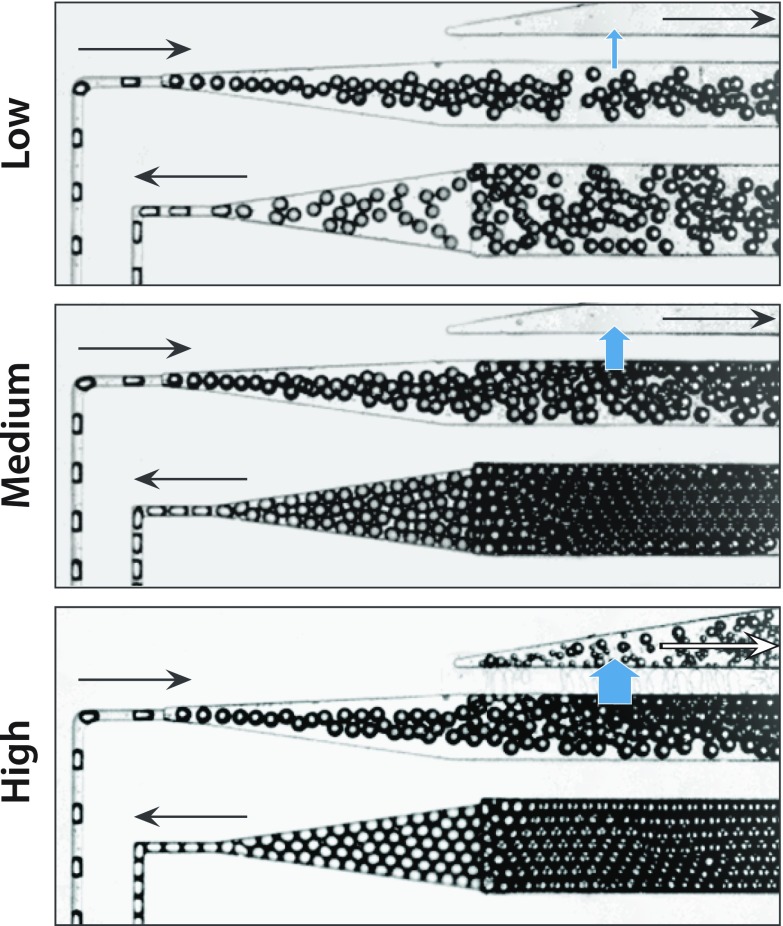
The oil extractor can remove controlled volumes of oil from an emulsion. To control the amount of oil removed, the outlet pressure of the oil extraction channel is controlled by gravity. Alternatively, for better reproducibility, a syringe pump in the withdrawing mode can be used to extract a controlled flow rate of oil from the extraction channel. For low draw rates, the droplets at the outlet are still unpacked, but for moderate and high draw rates, the droplets pack and order due to their monodispersity. High packing gives rise to plug flow, in which droplets travel through the delay line at equal speed.

A unique and valuable property of our oil extractor is that the amount of extraction is adjustable using a syringe pump to draw off oil to the desired fraction. To illustrate this, we form dilute emulsions and extract varying amounts of the oil (Fig. 2). At low extraction rates, little oil is removed and the droplets remain unpacked (Fig. 2, top). At moderate flow rates, the majority of oil is removed and droplets pack (Fig. 2, middle). At even higher flow rates, more oil is removed and droplets pack tightly; however, at these rates, pieces are also torn from the droplets (Fig. 2, bottom). This can be mitigated by fabricating even shorter extraction channels, although their hydrodynamic resistances must be carefully controlled to ensure the needed extraction rate with the available pressure drop through the extractor. Interestingly, for high extractions, we find that droplets adjacent to the oil extractor tend to coalesce. This may be due to shear-induced coalescence and could be a major source of the unintended merger during droplet reinjection from off-chip reservoirs, which is difficult to be observed due to the inability to image within syringes, needles, and tubing.

**FIG. 3.**
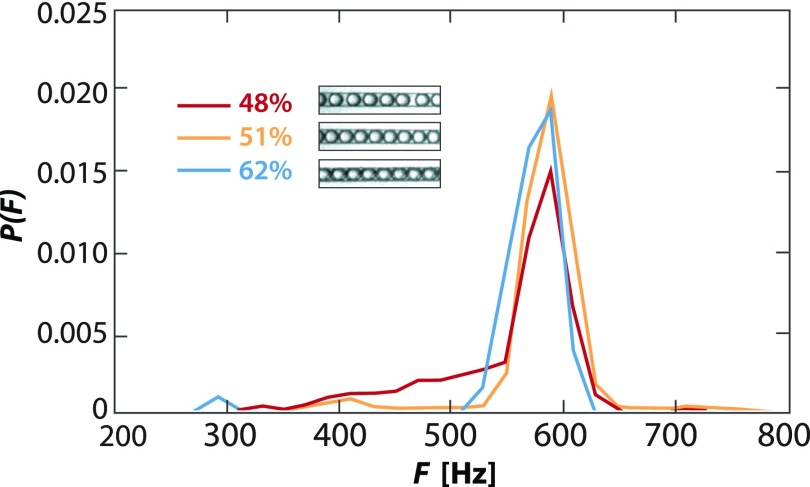
Packed droplets flow periodically through channels. As the droplets become more packed, they order due to their monodispersity, yielding periodic flow and a narrow distribution of frequencies. The major frequency of ∼570 Hz corresponds to two droplets touching while moving at the constant flow velocity.

Most droplet microfluidic devices are designed assuming periodic flow. This is essential for synchronizing streams for the pairwise merger^21^ or generating multiple emulsions with controlled numbers of cores and shells.^31^ The ability to extract a large fraction of oil from an emulsion is valuable because it allows initially aperiodic streams to be made periodic. To illustrate this, we measure the periodicity of droplets flowing through our device for varying degrees of extraction (Fig. 3). When we remove some oil (48% remaining aqueous), we observe a broad distribution of droplet frequencies. Many droplets are emitted at 520–620 Hz, corresponding to two touching droplets moving at constant velocity, but also observe a sizable fraction of low frequency events, corresponding to droplets spaced by random volumes of oil; these droplets lead to aperiodicity in the flow. As we extract more oil, the drops pack (51%) and the tail nearly vanishes, indicating good periodicity. As we increase extraction further (62%), we maintain good periodicity and observe even fewer low-frequency events.

**FIG. 4.**
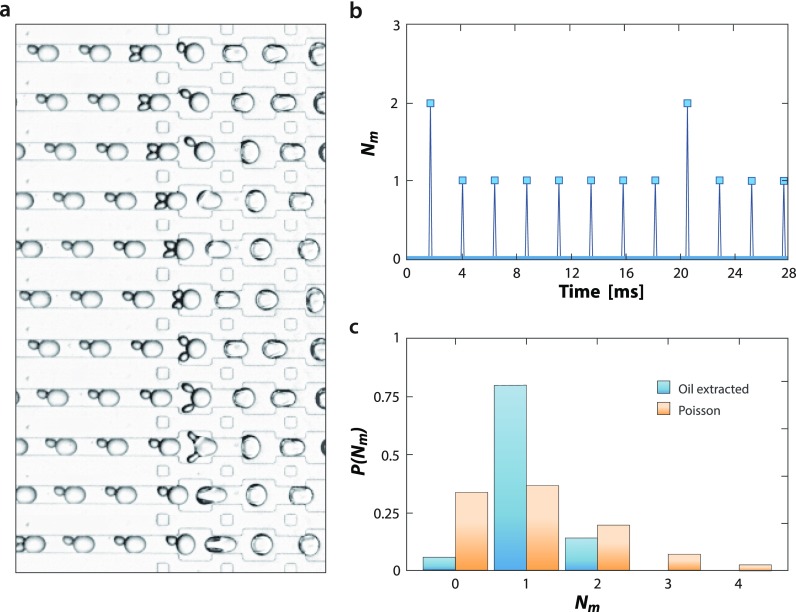
Droplet periodicity allows precision synchronization of streams for an efficient pairwise merger. (a) Packed, smaller droplets are synchronized with generated, larger droplets by adjusting flow rates on a merger device, and the pairs merged via electrocoalescence with salt-water electrodes. (b) Synchronization requires packed droplets be periodic and combined with the made droplets at equal frequency and phase, but small discrepancies can lead to “beat” patterns in which most events are pairwise mergers but some are three-way. *N_m_* is the number of smaller droplets merged with the incoming, larger droplets, that is, *N_m_* = 1 for pairwise merging. (c) Nevertheless, by making the incubated droplets periodic, a pairwise merger is achieved much more often than with random injection, given by a Poisson distribution. Blue bars show the distribution of pairing ratios with oil extraction obtained by analyzing 540 droplet merger events. Orange bars show the Poisson distribution with λ = 1.08.

The ability to pack droplets by extracting oil allows us to transform an aperiodic flow into a periodic one. This is valuable when droplets must be synchronized on a microfluidic device. To illustrate this, we synchronize the flow and the merger of two droplet streams, the first droplet made upstream on the device at a low volume fraction, packed by oil extraction, incubated for ∼30 s, and paired with the second stream formed by another droplet maker. We adjust the frequency of the second droplet maker to achieve near-synchronization of the streams and flow the pairs into a merger junction where the droplets are coalesced via an electric field applied by salt-water electrodes [Fig. 4(a)].^20^ The droplets are periodic, although the streams are not perfectly synchronized and, in particular, the incubated droplets enter at a slightly faster rate than the made droplets, resulting in ∼80% one-to-one fusions and ∼14% two-to-one [Fig. 4(b)]. Nevertheless, this is a major improvement over the merger of unpacked droplets which enter at roughly random intervals and thus are predicted to yield only ∼37% one-to-one fusions, assuming a Poisson distribution with the same average droplet number ratio [Fig. 4(c)]. The poor performance of randomly injected droplets in terms of the pairing ratio has also been experimentally confirmed (Fig. S1 in the supplementary material). This boost in the pairwise merger is important because unmerged droplets waste reagents and multiple mergers combine reactions, which can confound the results of an experiment. The ability to reliably synchronize droplet streams makes merging efficient and improves the data quality.

## IV. CONCLUSION

We have presented a device to efficiently extract oil from an emulsion and packed droplets together. This allows us to adjust the oil volume fraction between steps in a workflow and aperiodic streams to be synchronized with other operations such as merging, sorting, and double emulsion encapsulation. The ability to pack droplets yields plug-flow, in which all droplets move at identical speed, which is useful for incubating droplets for controlled times, so as to allow a cell to secrete a molecule or an enzyme to catalyze a reaction. The oil extractor affords a universal adaptor for connecting microfluidic components that do not operate under identical conditions and should thus enhance the reliability of multi-component devices. It should be valuable for applications requiring controlled delays, efficient mergers, or the generation of multiple emulsions with thin-shells.^32^

## V. SUPPLEMENTARY MATERIAL

See supplementary material for photomask design and additional experimental data.
